# Modern Methods of Transplantation

**Published:** 1914-01-31

**Authors:** 


### NEW DEVELOPMENTS IN MEDICINE.

### Modern Methods of Transplantation.

### AUTOPLASTY, HOMOPLASTY, AND HETEROPLASTY.

To transplant an organ or a tissue is not a modern innovation, however much it may seem so to those who are struck with admiration by the more daring methods of the American school. All plastic surgery, in a sense, is founded on the principle that with proper care and skilful adjustment it is possible to transplant living tissue and give it a foundation upon which it may flourish and incorporate itself with adjacent healthy tissue. But modern methods have progressed since the days of the Indian surgeons who made new noses by skin transplantation—a method still known as the Indian method of rhinoplasty. To-day we have a separate nomenclature for these processes. We speak of implantation—the restoration of a bit of tissue that has been separated from its fellows; of autoplasty—the transplantation of a tissue or an organ from one site to another in the same body; of homoplasty—the transplantation of tissues or organs from the same species; and of heteroplasty—the transplantation of organs or tissues from an individual of one species into the body of an individual belonging to another species. Much of the work so far done is experimental; but enough has been accomplished to prove that there are large possibilities in this field.

#### Medical Pioneers.

Jaboulay and Briau were the pioneers in vascular experimental work in this direction. In 1897 they succeeded in anastomosing vessels by means of living vessel tissue taken from the same animal. Gluck and Murphy improved on this method by invaginating methods, which simplified the technique and gave better results. Jeger, Stich, and Carrel still further improved the older technique, the latter's circular method being now generally adopted, though Dobrowolskaja's modification, in which the ends of the vessel openings are narrowed by plication, is used by many operators. Prothetic methods, in which the anastomosis is effected not by immediately suturing the transplanted vessel to the vessel in the patient's body, but a junction is made by means of a metal tube or ring, have been described by Payr, Fisher, Lepinasse, and Eisenstaedt among others. By the use of these methods, accompanied by the most careful aseptic precautions, excellent results have been obtained. Thus Ward succeeded in transplanting a piece of aorta taken from a guinea-pig into the carotid of a dog. Three months later the autopsy showed that the new channel had functioned perfectly, though the intima of the transplanted artery had been replaced by hyaline tissue. It is now generally accepted that heteroplastic transplantation, so far as vascular work is concerned, gives indifferent results compared with homoioplastic transplantation; in other words, the transplanted vessel is best taken from the patient's own body, and not from a dog or animal.

The first transplantation of the kidney was done by Ullmann in 1902. He succeeded in transplanting a dog's kidneys into the neck of the same dog, connecting the renal vessels with the carotid and the jugular according to Payr's method. The transplanted kidneys continued to function. Exner and Carrel repeated the experiment with similar results. Makas and Capelle went further and transplanted the kidneys into the thigh, connecting the renal vessels with the iliac vessels and inserting the ureter into the side of the bladder. Three weeks later the autopsy showed that the kidneys were unchanged. The experiments of Garre, Stich, and Borst and Enderlen have proved that such transplanted kidneys continue to function well, and that they seem to adapt themselves easily enough to their changed environment. In one case a dog lived for two years with a transplanted kidney after the other kidney had been extirpated. In one case a trial has actually been made on a human patient. This was a girl whose kidneys were almost functionless, and who was practically dying. Unger excised the kidneys of a monkey, and transplanted these into the patient's thighs, bringing the ureters out through the skin. The operation was done under the worst possible conditions, and the transplanted kidneys appeared to function to some extent, but thirty-two hours after the operation the patient died from uræmia. The autopsy showed that the kidney vessels were quite patent, and that there were no necrotic patches in the transplanted kidneys.

Transplantation of the thyroid is now a recognised operation of surgery, although the whole gland is rarely transplanted. Even for partial transplantation the conditions are difficult, since the vessels of this organ are thin and small, and end-to-end anastomosis is therefore a matter of extreme delicacy. Subcutaneous implantation is therefore preferred. In three cases of marked cretinism operated on by Borst and Enderlen, thyroid halves removed from goitrous patients were transplanted into the cretins, the axilla being chosen as site, and the thyroid vessels anastomosed with the brachial and cephalic artery and vein respectively. The operations were successful in so far that the transplanted organ lived, but no ultimate benefit was recorded. Probably the reason for this unsuccess lies in the tissue changes that the goitrous gland had undergone previous to its removal. The whole subject, however, is complicated by the results derived from recent experiments of Schone, who found that hereditary influences play some part in determining the functional activity of transplanted organs. Least successful so far has been the attempt to transplant the spleen.

On the other hand, skin-transplantation, better known as "skin-grafting," is a legitimate operation now carried out by every dresser in a general hospital. Subcutaneous implantation of the thyroid has given good results in many cases in clinical practice. Implantation of the pituitary has so far only been carried out in animals with indifferent results. Bone transplantation is now very generally and successfully used; the latest development is found in Albee's method, in which a piece of the patient's tibia is inserted into a groove cut in the spinous processes, in order to restore the integrity of the spine in cases of spinal caries.

### What Experiments have Taught.

Transplantations of cartilage, and even of whole joints, such as the knee, elbow, and shoulder joint, have been successfully effected on the human patient, while similar methods with tendons—tendon transplantation and tendon grafting—and nerves are recognised operations in orthopædic surgery. Fascia and mucous membrane transplantations have been successfully adopted in the treatment of hypospadias—in which a bit of excised appendix has done excellent service. Transplantation of muscles has been less successful. Attempts to restore the integrity of the stomach and intestinal tract by transplantation have so far been unsuccessful.

These details show that modern methods of transplantation have decided possibilities in their application to the human subject. Conditions are, of course, widely different in operating on a human patient from what they are in operating on an animal, and too much reliance must therefore not be placed on the results derived from vivisection experiments. But, at the same time, such experiments have demonstrated the practicability of the methods employed, and have shown that an organ as vascular and as highly developed as the ovary, thyroid, or kidney can be taken out of its original bed and inserted into a new site and still continue to function with success. That, at least, is a gain in practical knowledge, the application of which must be the concern of the surgeon in the future.

